# T-cell-derived TNF-α and a cluster of immunological parameters from plasma allow a separation between SARS-CoV-2 convalescent versus vaccinated elite athletes

**DOI:** 10.3389/fphys.2023.1203983

**Published:** 2023-06-22

**Authors:** Jana Palmowski, Sarah Kohnhorst, Pascal Bauer, Christian Puta, Simon Haunhorst, Kristina Gebhardt, Thomas Reichel, Christian Keller, Magdalena Huber, Hartmann Raifer, Karsten Krüger

**Affiliations:** ^1^ Department of Exercise Physiology and Sports Therapy, Institute of Sports Science, Justus Liebig University Giessen, Giessen, Germany; ^2^ Center for Tumor and Immunology, Institute for Systems Immunology, Marburg, Germany; ^3^ Department of Cardiology and Angiology, Justus-Liebig-University Giessen, Giessen, Germany; ^4^ Department of Sports Medicine and Health Promotion, Friedrich Schiller University Jena, Jena, Germany; ^5^ Center for Sepsis Control and Care (CSCC), Jena University Hospital, Friedrich-Schiller-University Jena, Jena, Germany; ^6^ Center for Interdisciplinary Prevention of Diseases Related to Professional Activities, Jena, Germany; ^7^ Institute of Virology, Philipps University Marburg, Marburg, Germany

**Keywords:** T-lymphocyte, SARS-CoV-2, vaccination, convalescence, elite athletes

## Abstract

Guidelines for medical clearing after SARS-CoV-2 infection in elite athletes do not include T-cell immunity aspects despite its relevance in the course of COVID-19 disease. Therefore, we aimed to analyze T-cell-related cytokines before and after *in-vitro* activation of CD4^+^ T-cells. We sampled professional indoor sports athletes at medical clearing after SARS-CoV-2 infection obtaining clinical, fitness data, and serological data including CD4^+^ T-cell cytokines. All data were analyzed by principal component analysis and 2 × 2 repeated measures ANOVA. CD4^+^ T-cells were sampled for cell culture activation with anti-CD3/anti-CD28 tetramers. At medical clearing, CD4^+^ T-cells from convalescent athletes secreted increased levels of TNF-α 72 h after *in-vitro* activation compared to vaccinated athletes. IL-18 levels in plasma were elevated and a cluster of parameters differentiated convalescent from vaccinated athletes by 13 parameters at the timepoint of medical clearing. All clinical data indicate infection is resolved, while increased TNF-α may reflect altered proportions of peripheral T-cells as a hangover of infection.

## 1 Introduction

Indoor team sports players have a high transmission risk for severe acute respiratory syndrome coronavirus 2 (SARS-CoV-2) (Jones et al., 2021; Pauser et al., 2021) as indoor facilities ameliorate inhalation of respiratory droplets (and thus SARS-CoV-2), which are emitted by talking, coughing, sneezing, and breathing, contributing to viral spread (Tang et al., 2021; Mutsch et al., 2022). Acute coronavirus disease 2019 (COVID-19) caused by SARS-CoV-2 can be characterized by classical respiratory symptoms such as cough and fever in the mild course of the disease in young patients (Leung, 2021). The severity of acute coronavirus disease 2019 (COVID-19) caused by SARS-CoV-2 is mostly mild in young professional athletes and physically active adults (Sallis et al., 2021; Krzywański et al., 2022). However, the infection itself poses a risk, especially for athletes, if they return to sport too early without the infection being completely resolved. For instance, the incidence of cardiac complications such as myocarditis after COVID-19 ranges internationally from 1.1%–2.3% among competitive athletes (Halle et al., 2021). Myocarditis is difficult to diagnose as symptoms are non-specific (Daniels et al., 2021). Moreover, dysregulation of T-cell subsets is a key factor in myocardial inflammation (Wang and Han, 2020). The rate itself is relatively low, but myocarditis is one of the leading causes of sports-related sudden cardiac arrest in Germany (Bohm et al., 2021). Therefore, medical clearing after infection is given a special clinical focus in competitive sports (Phelan et al., 2020). The analysis of T-cell immunology may strengthen sufficient medical clearing.

Mild courses of disease in young elite athletes are influenced by CD4^+^ T-cell type 1 (Th1) immune response protecting the infected from developing severe COVID-19 disease (Chen et al., 2022; Krzywański et al., 2022; Moss, 2022). Th1 differentiation is associated with the secretion of the signature cytokines, such as Interferon-gamma (IFN-γ) and tumor necrosis factor-alpha (TNF-α) (Raphael et al., 2015). SARS-CoV-2–specific T-cells of previously infected patients predominantly produce Th1 cytokines (Weiskopf et al., 2020). Higher polyfunctional TNF-α+ IFN-γ+ T-cell frequency was also found after vaccination, but it is transient decreasing significantly after 6 months (Guerrera et al., 2021).

While expert guidelines for medical clearing after SARS-CoV-2 infection have been established based on subjective psychological and cardio-pulmonary examinations, objective immunological aspects other than typical clinical blood markers have not yet been considered as parameters for medical clearance and are currently unknown (Elliott et al., 2020; Wilson et al., 2020). The relevance of T-cell immunity implies the need for analyzing the athletes for T-cell-related cytokines in plasma and T-cell-activation cytokines within the context of other common clinical parameters. Therefore, the aim of our study was to analyze T-cell-related cytokines before and after *in-vitro* activation of CD4^+^ T-cells sampled from convalescent and vaccinated indoor sports athletes at medical clearing.

## 2 Methods

### 2.1 Participants

All professional athletes are part of the cross-sectional, single-center pilot study obtaining clinical and fitness data from athletes in the handball and ice-hockey pre-seasons (Bauer et al., 2022). Inclusion criteria were participation in professional indoor sports, no chronic diseases, age between 18 and 30 years, and full physical fitness at the immunological testing. All participants were part of the mentioned single-center pilot study and gave additional consent to their participation in this study. Either COVID-19 convalescent athletes (Con) at the time of medical clearing or non-infected controls (Vac) at the time of medical clearing pre-season were included. Due to the high vaccination rates in professional sports, all non-infected athletes were fully vaccinated. Participating athletes were 28.2 ± 6 years with a height of 189.4 ± 5.4 cm and a weight of 95.1 ± 9.3 kg. Their body mass index was 26.5 ± 1.8 kg/m^2^. Subjects were experienced professional athletes with a mean training time of 1,152 min per week.

### 2.2 Study-design

**FIGURE 1:**
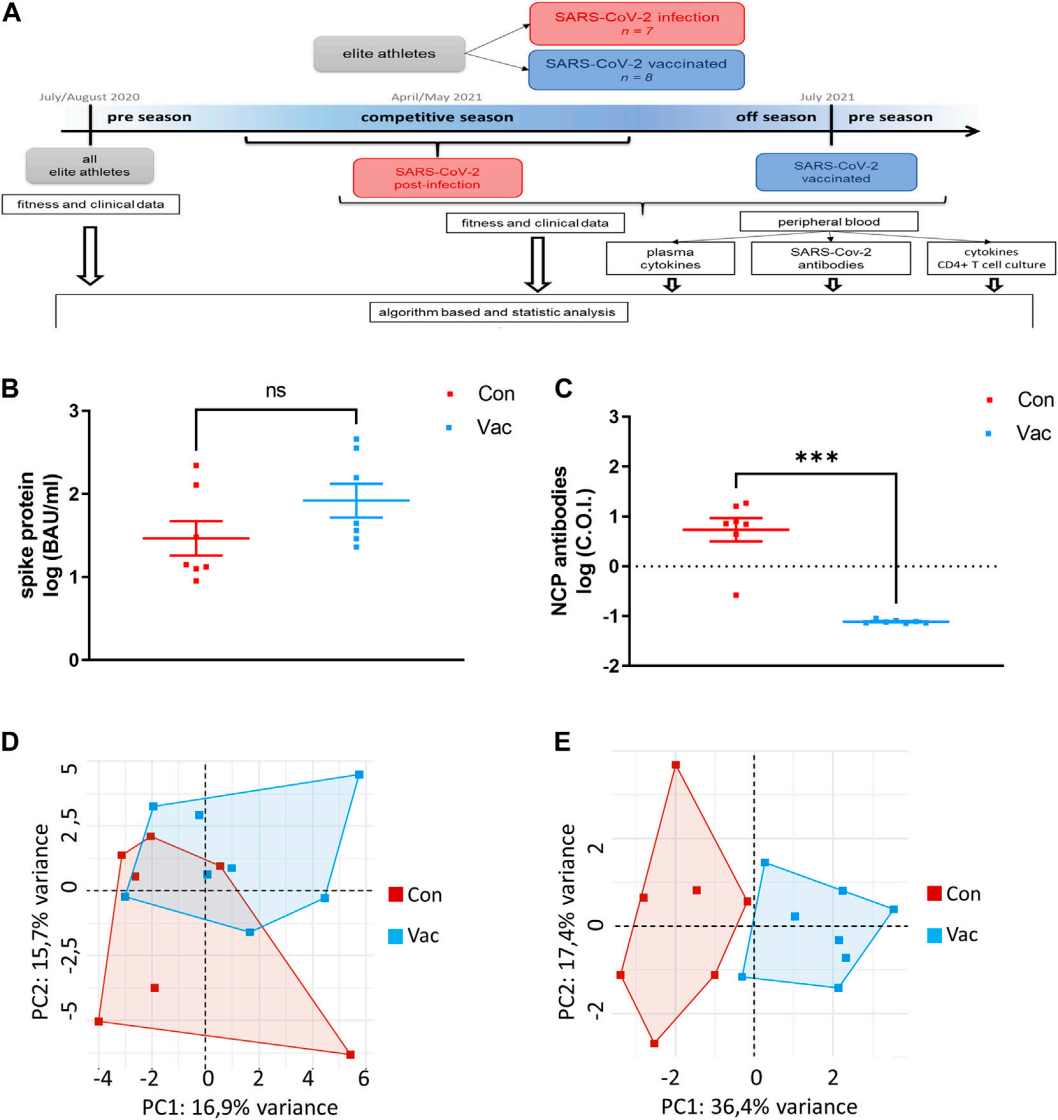
Workflow of this Study **(A)**. Concentrations of IgG antibodies against Spike were measured in plasma **(B)**. Total nucleocapsid protein-specific antibodies (NCP) were measured in plasma. One athlete belonging to Con did not show NCP antibodies. C.O.I. stands for cut-off-index which was set at on for this test. The dashed line indicates the detection limit **(C)**. PCA were calculated including all immunological and clinical parameters **(D)** as well as excluding all parameters, which show a statistical difference of *p* ≤ 0.1 **(E)**. For all parts of the figure, Con means cells were isolated from convalescent athletes. Vac means T-cells were isolated from non-infected, COVID-19-vaccinated athletes (Con *N* = 7, Vac *N* = 8). Significance was determined by the Mann-Whitney test (*p* < 0.05, ***p < 0.001). Data are shown as mean ± SEM.

We used an experimental study design performing cell culture analysis to obtain immunological data. This study arm was integrated into an observational controlled single-center pilot study design obtaining a broad panel of hematological and cardiovascular parameters as part of pre-season medical clearing (Bauer et al., 2022). This data was integrated into our systemic analysis of the immunological study arm. This additional study arm was approved by the Institutional Review Board of the Justus-Liebig-University Giessen (AZ183/20, 16 March 21) following the ethical guidelines of the declaration of Helsinki. Written informed consent was provided by all participants. Convalescent athletes were invited to the laboratory 14 days after the positive polymerase chain reaction (PCR) test result following the German quarantine regulations and available guidelines for medical clearing (Schellhorn et al., 2020). Control athletes were investigated following the same procedure after their off-season to not interfere with exercise-related immune disruption by high-intensive exercise training. For an overview of our workflow see Figure 1A.

### 2.3 Immunological data

For immunological analysis, CD4^+^ T-cells, serum, and plasma were obtained from seven convalescent and eight non-infected fully vaccinated men. Fully vaccinated refers to the German definition at the time from May to August 2022; meaning two doses for BNT162b2 (Pfizer-BioNTech), two doses for ChAdOx1-S (Oxford-AstraZeneca), and one dose for Ad26.COV2.S COVID-19 (Johnson and Johnson/Janssen). T-cells were isolated from peripheral blood via density gradient centrifugation and subsequent negative selection using EasySep™ magnetic particles (Stemcell Technologies, Vancouver, BC, Canada) as described by our group before (Palmowski et al., 2021). Isolated CD4^+^ were activated with anti-CD3/anti-CD28 stimulating tetramers (Stemcell Technologies, Vancouver, BC, Canada) or remained non-stimulated as controls. Cultures were maintained in IL-2 (Stemcell Technologies, Vancouver, BC, Canada) containing medium supplemented by 10% human AB-serum (Pan-Biotech, Aidenbach, Germany) for 72 h until harvest. Cell number, viability, and cell diameter were measured by CASY^®^ Cell Counter (OLSOmni Life Science, Bremen, Germany).

Frozen plasma was thawed, labeled using LEGENDplex Multi-Analyte Flow Assay kit “Human Inflammation Panel 1” (BioLegend, San Diego, CA), and measured by flow cytometry (BD FACSAria™ III flow cytometer). Raw data were analyzed by LEGENDplex™ data analysis software (BioLegend, San Diego, CA). Antibody titers were analyzed using the Elecsys anti-SARS-CoV-2 assay on the cobas 601 (Roche, Rotkreuz, Switzerland). Frozen supernatants were thawed and labeled for IFN-γ, IL-2, IL-4, IL-5, IL-6, IL-10, IL-13. IL-17A, and TNF-α using a Luminex assay (Bio-Techne Ltd., Abingdon, Oxon, United Kingdom) and following the manufacturer´s instructions. Plasma was analyzed by the Luminex MAGPIX system (Luminex Corporation, Austin, TX, United States).

### 2.4 Clinical and fitness data

All athletes completed the pre-participation questionnaire of the European Federation of Sports Medicine Associations including questions related to health, medication, supplementation, exercise history, and exercise volume. Clinical and fitness data was sampled during the pre-season of handball and ice hockey, and return-to-sport as described before (Bauer et al., 2022). As athletes completed electrocardiographic examinations including a stress test on the bike until subjective exhaustion as part of the medical clearing. Performance was calculated as the participants’ wattage at exhaustion in relation to their body weight. As part of the clinical examination laboratory blood parameters, such as blood sugar, HbA1c, electrolyte, iron metabolism, coagulation, liver, kidney, muscular enzyme, lipid, and thyroid hormone status were collected. All participants were attested with full physical fitness at the immunological testing.

### 2.5 Statistical analysis

Bioinformatics analysis was performed using RStudio running R software (4.1.2). All data are presented as mean ± standard deviation (SD). Data were tested for normal distribution by the Shapiro-Wilk test. To determine the effect of SARS-CoV-2 infection at the time of immunological testing, Wilcoxon signed-rank tests were conducted with all pre- and post-clinical, immunological, and fitness data of all 15 athletes after infection or vaccination. To visualize the differences between the SARS-CoV-2 infected and SARS-CoV-2 vaccinated group we performed a PCA (package factoextra) with 59 clinical and immunological parameters and one with clinical and immunological parameters that had a statistical difference of *p* ≤ 0.1. R-based graphs were created with packages ggplot2 3.4.1 and ggpubr 0.6.0.

Cell culture results were analyzed by a 2-by-2 repeated-measures ANOVA with activation status as the within factor (control cells vs. activated cells) and group (Con vs. Vac) as the between factor. Bonferroni post-hoc test was used. For analysis and visualization we used GraphPad Prism version 5 (San Diego, CA, United States). Data were shown as mean ± standard deviation. The same statistics were also applied for the comparison between baseline and follow-up clinical parameters changing the within factor to time. The significance level was set at *p* < 0.05.

## 3 Results

### 3.1 Immunological parameters

Analysis of SARS-CoV-2 specific antibodies show no significant differences between Con and Vac in spike-specific plasma IgG antibody titers (Figure 1B). However, in contrast to Con, Vac are non-reactive against the nucleocapsid protein (NCP) (Figure 1C). One athlete belonging to Con did not show NCP antibodies. At the time point of our immunological analysis, there was no significant difference in pro-inflammatory plasma cytokines except for IL-18 (Table 1). IL-18 plasma level was increased in Vac compared to Con.

**TABLE 1:** Pro-inflammatory cytokines in plasma at the time point of immunological analysis. Plasma was measured using LEGENDplex Multi-Analyte Flow Assay kit “Human Inflammation Panel 1” (BioLegend, San Diego, CA) and flow cytometry (BD FACSAria™ III flow cytometer). Wilcoxon signed-rank tests were conducted. (*p* < 0.05). Data are shown as mean ± SD. Significant findings are highlighted in bold letters.

	Con		Vac		
	Mean [pg/ml]	SD [pg/m]	Mean [pg/ml]	SD [pg/ml]	p-value
IL-1β	12.57	5.54	13.62	4.44	0.424
IFN-α2	18.42	12.37	21.25	17.47	0.897
IFN-γ	7.07	5.43	6.30	4.17	1.000
TNF-α	25.39	23.71	34.75	34.08	0.523
MCP-1	20.23	17.63	24.11	23.28	0.698
IL-6	8.87	3.87	11.15	7.45	0.436
IL-8	38.70	11.95	60.39	35.22	0.369
IL-10	10.09	7.65	13.50	9.04	0.513
IL-12p70	7.22	4.13	6.64	1.84	0.887
IL-17A	2.98	1.93	2.87	1.41	0.796
IL-18	244.70	64.58	368.75	100.38	**0.030**
IL-23	9.66	8.05	20.26	25.59	0.887
IL-33	190.34	166.67	160.58	105.87	0.898

### 3.2 Principal component analysis

Principal component analysis revealed that all immunological and clinical parameters allow a slight separation between Con and Vac by systemic analysis at the time of immunological testing, while 13 parameters separated infected from vaccinated samples significantly (Figures 1D, E). These were: Creatinine, glomerular filtration rate (GFR), glutamic oxaloacetic transaminase, potassium, creatine kinase, glycated hemoglobin (HbA1c), triiodothyronine, ferritin, myoglobin, cardiovascular parameters (diastolic aortic pressure, mean aortic pressure, and diastolic brachial blood pressure), and TNF-α derived from activated T-cell culture (Table 2).

**TABLE 2:** Selected parameters at the time point of immunological testing that were identified by PCA. All parameters are measured in plasma despite TNF-α that was measured in cell culture supernatants. Data are shown as mean ± SD. Significant findings are highlighted in bold letters.

	Con		Vac		
	Mean	SD	Mean	SD	p (post)
Diastolic aortic pressure	74.43	8.89	64.63	6.65	**0.036**
Mean aortic pressure	85.86	7.66	77.33	6.26	**0.028**
Creatinine	0.90	0.08	1.03	0.07	**0.015**
Glomerular filtration rate	105.81	9.24	94.49	8.64	**0.049**
Ferritin	206.14	52.27	126.25	92.03	**0.032**
Triiodothyronine	3.53	0.20	3.21	0.31	**0.040**
Creatine kinase	117.29	41.05	371.13	305.08	**0.013**
Myoglobin	65.29	92.10	50.00	27.09	**0.042**
TNF-alpha	6083.75	122.94	5845.88	127.15	**0.007**
Diastolic brachial blood pressure	72.86	9.25	64.13	5.84	0.064
Glycated hemoglobin	5.43	0.27	5.13	0.35	0.071
Potassium	4.16	0.26	3.85	0.33	0.080
Glutamic oxaloacetic transaminase	19.00	5.00	28.13	12.24	0.091

### 3.3 Activated cell culture

**FIGURE 2:**
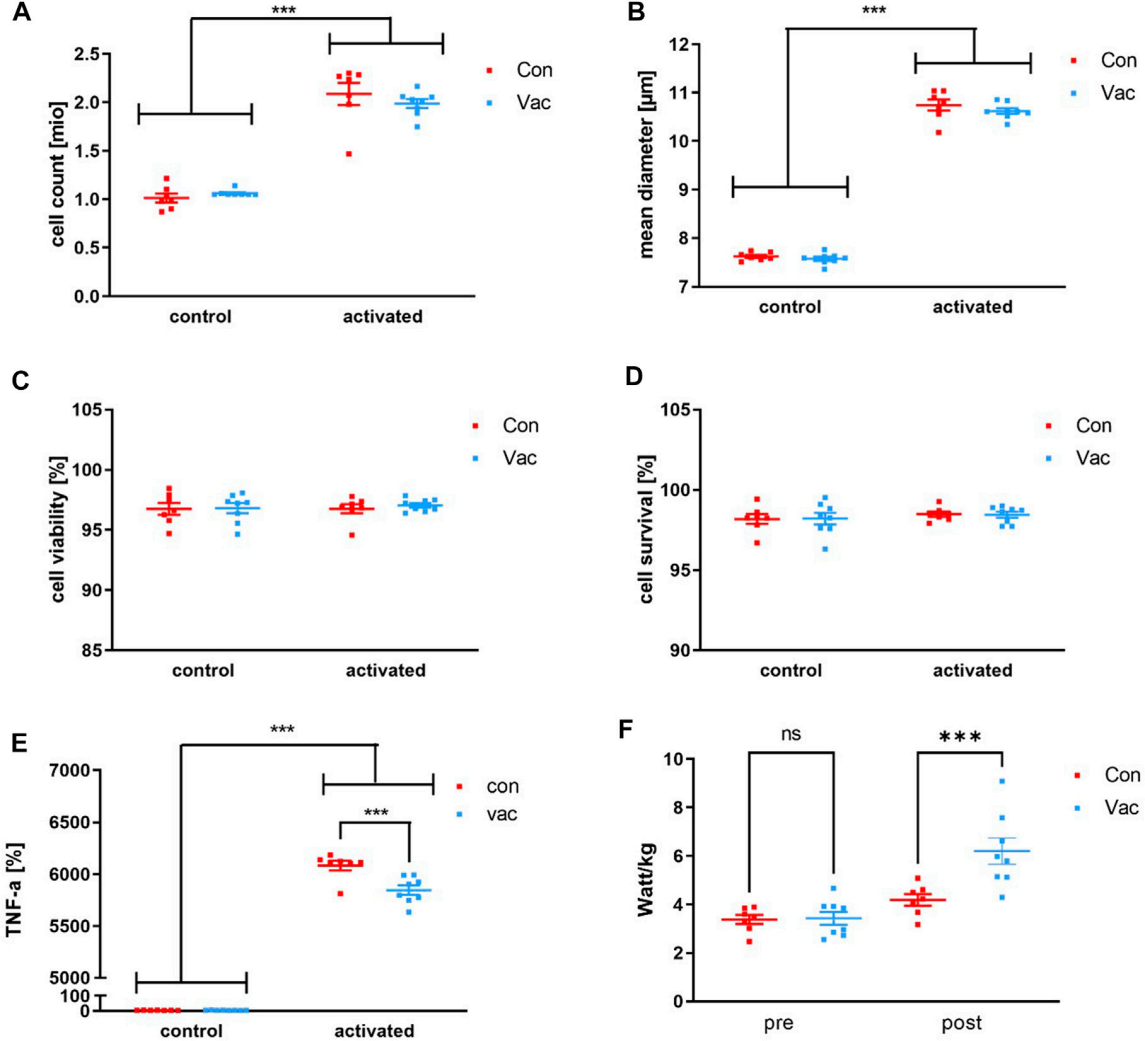
T-cell count **(A)**, mean diameter **(B)**, viability **(C)**, and survival **(D)** after activation of 1 million CD4^+^ T-cells using anti-CD3/anti-CD28 tetrameres. T cell-derived TNF-α levels were measured after 72 h post activation of 1 million CD4^+^ T-cells with anti-CD3/anti-CD28 tetramers (Stemcell Technologies, Vancouver, BC, Canada) or after 72 h in non-activated controls **(E)**. Performance was calculated from the ECG stress test as the participants’ wattage at exhaustion in relation to their body weight **(F)**. Cell culture supernatants such as TNF-α were analyzed using a commercially available human Magnetic Luminex Assay (Bio-Techne, Abingdon, United Kingdom) by Magpix Luminex Instrument (Luminex Corp, Austin, TX, United States). Significance was determined by a repeated-measures 2 × 2 ANOVA and Bonferroni posttest. TNF-α levels were significant for activation status, group, and interaction, with a significant difference between Con and Vac for post-activation TNF-α levels showed by Bonferroni posttest (*p < 0.05, ***p < 0.001). Con refers to T-cells that were isolated from convalescent athletes, and Vac refers to T-cells that were isolated from vaccinated athletes (Con *N* = 7, Vac *N* = 8). Data are shown as mean ± SEM.

A significant activation-driven effect was detectable for total cell count and cell diameter of the *in vitro* experiments, but not for viability and survival. We calculated cell survival via the formula: Viability = (100%− viability before culture) + viability after cell culture. Con and Vac measurements were comparable 72-h post-activation (Figures 2A–D; Supplementary Table S1). Likewise, a significant effect for all measured T-cell signature cytokines was found after activation, namely, for IFN-γ, IL-2, IL-4, IL-5, IL-6, IL-10, IL-13, IL-17A, and TNF-α (Supplementary Table S1). However, only the levels for TNF-α significantly differed between Vac vs. Con. Supernatants from Con CD4^+^ T-cells showed increased TNF-a levels compared to the Vac after the Bonferroni posttest with *p* > 0.001 (Figure 2E).

### 3.4 Clinical parameters

All laboratory parameters were within the physiologic range. Two-way ANOVA showed a significant effect for time for aortic diastolic and systolic aortic blood pressure for all athletes, Vac and Con, at return-to-sport. Blood pressure was higher at immunological sampling. An effect for time was also found for lactate dehydrogenase with significantly lower mean values both at immunological sampling. A significant effect for the condition was found on HbA1c and free triiodothyronine and a significant interaction of time and condition was shown for potassium, magnesium, and creatinine (Supplementary Table S2).

### 3.5 Fitness parameters

Exercise testing revealed a lower performance/weight ratio for Con compared to Vac. Of note, the individual performance does not fall below pre-season performance after quarantine (Figure 2F).

## 4 Discussion

The present work evaluated T-cell-related cytokines before and after *in-vitro* activation of CD4^+^ T-cells sampled from convalescent and vaccinated indoor sports athletes to assess signatures of clinical and immunological parameters in elite athletes who have passed a SARS-CoV-2 infection. There were no elevated levels of inflammatory cytokines in the plasma of Con compared to Vac. Also, no immune disturbance in Con T-cells in terms of activation with anti-CD3/anti-CD28 tetrameres was found. *In vitro* cultivated CD4^+^ T-cells showed the expected signs of T-cell activation such as increased cell number, cell diameter, and increased production of T-cell signature cytokines. However, there are indications that can be interpreted as residual infection indicators, such as an increased TNF-α production in activated T-cells and reduced performance.

### 4.1 Increased TNF-α production of CD4^+^ T-cells in Con athletes after activation

After T-cell activation, TNF-α production was significantly increased by cells of Con compared to Vac, whereby overall T-cell number, mean cell viability, total cell count, and cell diameter in the *in vitro* experiments were comparable between both groups. Mobilization of peripheral T-cells may have influenced our *in vitro* cell culture results. Increased TNF-α level in Con after activation may be biased by a type 1 differentiation as TNF-α is a classical type 1 cytokine and indicates a higher pro-inflammatory response (Mahnke et al., 2013). TNF-α is produced by type 1 cells, which are possibly still present in greater numbers in peripheral blood after an effective immune response and release TNF-α after activation (Rosendahl Huber et al., 2014). In line with that, it was shown for other convalescent patients that SARS-CoV-2–specific T-cells predominantly produce Th1 cytokines (Weiskopf et al., 2020). At the same time, the difference in TNF-α level could also be caused by metabolic disturbance of T-cell glycolysis in Vac. *In vitro* studies with isolated CD4^+^ have shown, glycolytic inhibitor 2-deoxy-glucose reduces TNF-α production (Renner et al., 2015). Overall, the function of CD4^+^ T-cells after activation is not reduced after SARS-CoV-2 infection in athletes. Their peripheral cells react to tetramere activation more sensitively than Vac CD4^+^ T-cells.

### 4.2 Increased pro-inflammatory IL-18 in Vac

An elevated level of pro-inflammatory plasma cytokine was found for IL-18 in Vac participants compared to Con. IL-18 is secreted by various cell types such as macrophages, B cells, dendritic cells, keratinocytes, and even type 2 muscle fiber (Mee et al., 2000; Plomgaard et al., 2005; Dinarello et al., 2013). The cytokine is known for its pro-inflammatory properties. It is activated in an NLPR3 inflammasome-dependent manner and enhances Th1 cell activation (Mantovani et al., 2019). We measured similar IL-18 levels for Con athletes compared to other studies that measured IL-18 in healthy young men (Leick et al., 2007; Khakroo Abkenar et al., 2019). So, we conclude, Con are fully recovered in terms of inflammatory mediators available in plasma. The elevated plasma IL-18 level in Vac could be exercise-related because this cytokine is highly sensitive to resistance exercise training and detraining (Nikseresht et al., 2016; Khakroo Abkenar et al., 2019). Young men showed significantly higher plasma IL-18 accompanied by higher IL-1ß levels immediately after acute high-intensity exercise and after 12 weeks of high-intensity exercise training (Khakroo Abkenar et al., 2019). As we found no increase in IL-1ß plasma levels and Vac were included after the off-season, an effect of high-intensity exercise training on IL-18 level is unlikely. There may be an effect of detraining on Vac that were included after their full off-season and not merely 14 days post-quarantine. Type 2 muscle fiber number can be maintained within the first initial weeks of detraining. However, a shift to more oxidative fibers in strength athletes is found after 8 weeks of detraining (Bruton, 2002). Potentially this loss of type-2 muscle fiber is attributed to higher IL-18 levels in Vac (Nikseresht et al., 2016). Of note, IL-18 levels were also increased in mRNA-vaccinated patients that acquired myopericarditis and psychiatric disorders such as major depression and panic disorder (Kokai et al., 2002; Won et al., 2022).

### 4.3 Residual infection markers within normal range differentiate Con and Vac athletes

While systemic inflammation of Con athletes based on pro-inflammatory cytokines was resolved, there may still be other residual signs of infection, as indicated by the systemic analysis at medical clearing for ferritin, GFR, and blood pressure. Increased ferritin is common for viral infections (Slaats et al., 2016). Ferritin represents an acute phase protein that is elevated both generally in viral infections and specifically in COVID-19 (Vlahakos et al., 2021). Most likely, residual ferritin may be caused by the physiological apoptosis of iron-depleted immune cells (Arigony et al., 2013). GFR might be attributed to increased blood pressure. In context, the other study arm including professional athletes has found an increased heart rate-corrected augmentation index for Con reflecting lower arterial stiffness (Bauer et al., 2022). Interpreting blood pressure results, one should include emotional adrenergic-related distress caused by quarantine (di Cagno et al., 2020) and its influence on T-cells. Immunologically, sympathetic stress also regulates T-cell function via the beta2-adrenergic receptor (β2AR) with a potential to alter T-cell activation (Fan and Wang, 2009). Under psychological stressors, humans mobilize particularly peripheral T-cells expressing CXCR3 and CCR5 that sensitively react to chemotactic signals of inflamed endothelial cells (Bosch et al., 2003). However, this mobilization likely is not able to solely explain differences in T-cell activation as blood pressure of Con and Vac both increased across time as indicated by the 2-by-2 ANOVA results.

### 4.4 Lower cardiovascular fitness level in Con

Our data shows the Con’s cardiopulmonary performance in watt/kg was lower compared to Vac, though still higher than at pre-season. The COVID-19 detraining effect was not below baseline levels at pre-season. Lower performance levels come as no surprise, as performance-limiting factors, for instance, muscular capillarization, are known to decrease in only 2 weeks of detraining even in healthy athletes (Bruton, 2002). Performance decline has been mainly attributed to changes in hemodynamics and neuromuscular adaptations, such as maximal oxygen consumption or muscle strength at maintained muscle mass (Chen et al., 2022). In line, a recent COVID-19 study including soccer players also found a decline in high-intensity acceleration performance indicating a lower anaerobic threshold (Savicevic et al., 2021).

### 4.5 Limitations

Current vaccines use the spike protein as a target antigen structure, consequently, a spike-specific immunity is induced. After the infection, the immune response is directed against all viral proteins and not only against the spike protein. Thus, only people who were infected with the virus should have NCP-specific antibodies (Jalkanen et al., 2021). We assumed no unknown infection in the vaccinated control group. However, one Con was non-reactive against NCP, therefore it cannot be ruled out there may be also Vac who had an infection with COVID-19 earlier without building NCP. Further, we cannot exclude, that differences between Con and Vac result from a selection bias. Athletes for this study were selected from the same teams indicating that naturally infected athletes (Con) may have lower immune competence compared to non-infected athletes playing on the same team (Vac).

Unfortunately, our study lacks a control group of non-vaccinated elite athletes and the sample size is low due to the laborious experimental design. This suggests caution when interpreting the findings. Due to the high vaccination rate over the course of the study and a high number of early infected athletes in indoor sports, non-vaccinated elite athletes were not available at the time of sampling in 2021. Although all athletes participating had a reduced physical activity level before the immunological study part, groups may differ by activity level at least 14 days before measurement as convalescent athletes are more limited to physical activity due to quarantine regulations. Interpreting our result, one should take caution of the different training phases the athletes were at before their time off practice. This may have influenced the differences in IL-18 plasma levels. *In vivo*, activation of T-cells would likely be influenced by higher IL-18 levels, while our *in vitro* cell culture was supplemented by non-autologous human AB-serum causing cytokine-wise equal cell culture conditions. IL-18 would likely induce Th1 T-cells after activation (Robinson and O’Garra, 2002).

## 5 Conclusion

T-cell-related immunological aspects other than typical clinical blood markers have not been investigated at medical clearance in Con after COVID-19 yet. Overall, caution is needed in interpreting our results given the small sample size. Our data shows expert-guided medical clearing after COVID-19 is sufficient to bring immunological parameters back to baseline. Con even have a higher inflammatory response to T-cell activation compared to Vac, indicated by higher TNF-a levels. If this is caused by Th1 response and a transient residual sign of infection, should be investigated in future studies. Despite the resolution of inflammation, athletes and coaches should take into account that athletes’ T-cells may act more sensitive to immune activation after SARS-CoV-2 infection up to medical clearance.

## Data Availability

The original contributions presented in the study are included in the article/Supplementary Material, further inquiries can be directed to the corresponding author.
