# Supplementary material for: T-cell-derived TNF-α and a cluster of immunological parameters from plasma allow a separation between SARS-CoV-2 convalescent versus vaccinated elite athletes
Source: Front Physiol. 2023 Jun 22;14:1203983. doi: 10.3389/fphys.2023.1203983 (PMC10324374; doi:10.3389/fphys.2023.1203983)
Supplement: Supplementary file 1 [file Table1.DOCX]

Supplementary Material

Brief research report: T cell-derived TNF-a and a cluster of immunological parameters from plasma allow a separation between SARS-CoV-2 convalescent versus vaccinated elite athletes

Jana Palmowski^1^, Sarah Kohnhorst^2^, Pascal Bauer^3^, Christian Puta^4^, Simon Haunhorst^4^, Kristina Gebhardt^1^, Thomas Reichel^1^, Christian Keller^5^Magdalena Huber^2,^ Hartmann Raifer^2†^, Karsten Krüger^1†*^

^1^Institute of Sports Science, Department of Exercise Physiology and Sports Therapy, Justus Liebig University Giessen, Giessen

^2^Institute for Systems Immunology, Center for Tumor und Immunology, Marburg

^3^Department of Cardiology and Angiology, Justus-Liebig-University Giessen, Giessen

^4^Department of Sports Medicine and Health Promotion, Friedrich Schiller University Jena, Jena

^5^Institute of Virology, Philipps University Marburg, Marburg, Germany.

*** Correspondence:**Corresponding Author
[Karsten.Krueger@sport.uni-giessen.de](mailto:Karsten.Krueger@sport.uni-giessen.de)

^†^ These authors jointly supervised this work

# Supplementary Figures and Tables

## Supplementary Tables

**Supplementary Table 1.** Overview on cell culture parameters and signature cytokines of T-cells at control condition and after 72h-activation with anti-CD3/antiCD28 tetrameres. Cells were sampled from convalescent and non-infected, Covid-19-vaccinated athletes (Con N=7, Vac N=8). Data was analyzed using a 2x2 ANOVA with group factor cell culture condition and within factor athlete’s infection status. Significant results (p<0.5) are highlighted in bold letters.

|  |  | **Con** | | | | **Vac** | | | |  |  |  |
| --- | --- | --- | --- | --- | --- | --- | --- | --- | --- | --- | --- | --- |
|  |  | Control | | Activated | | Control | | Activated | | p | | |
|  |  | Mean | SD | Mean | SD | Mean | SD | Mean | SD | Infection status | Condition | Interaction |
| **T-cell-signature cytokines** | |  |  |  |  |  |  |  |  |  |  |  |
|  | IFN-γ (pg/µl) | 12.57 | 5.04 | 53297.08 | 3778.37 | 38.12 | 26.39 | 47939.38 | 8686.14 | 0.0909 | **0.0004** | 0.3072 |
|  | IL-10 (pg/µl) | 2.09 | 0.68 | 3068.75 | 1196.87 | 2.16 | 0.46 | 3535.71 | 1120.94 | 0.0805 | **< 0.0001** | 0.0806 |
|  | IL-13 (pg/µl) | 114.00 | 27.98 | 2346.02 | 837.96 | 148.10 | 21.54 | 2657.23 | 250.02 | 0.2072 | **< 0.0001** | 0.2456 |
|  | IL-17A (pg/µl) | 3.77 | 1.61 | 8653.63 | 2475.25 | 4.59 | 0.63 | 5724.32 | 4229.06 | 0.148 | **< 0.0001** | 0.3565 |
|  | IL-2 (pg/µl) | 8899.77 | 3052.28 | 14734.40 | 4450.18 | 12224.0 | 2153.46 | 16458.71 | 2826.81 | 0.3568 | **0.0001** | 0.7464 |
|  | IL-4 (pg/µl) | 80.00 | 68.84 | 1271.35 | 903.29 | 41.23 | 3.85 | 1327.22 | 515.95 | 0.3623 | **0.0004** | 0.3072 |
|  | IL-5 (pg/µl) | 19.39 | 40.23 | 1763.16 | 852.02 | 6.78 | 5.29 | 2138.29 | 485.38 | 0.2659 | **< 0.0001** | 0.2463 |
|  | IL-6 (pg/µl) | 1.21 | 0.27 | 2415.19 | 1014.03 | 1.70 | 0.36 | 2543.71 | 602.23 | 0.5441 | **< 0.0001** | 0.5451 |
|  | TNF-α (pg/µl) | 4.41 | 1.17 | 6083.75 | 122.94 | 4.96 | 1.01 | 5845.88 | 127.15 | **0.0028** | **< 0.0001** | **0.0028** |
|  | **Cell culture parameters** |  |  |  |  |  |  |  |  |  |  |  |
|  | Cell count [mio.] | 1.014 | 0.119 | 2.088 | 0.299 | 1.061 | 0.032 | 1.988 | 0.127 | 0.677 | **< 0.0001** | 0.2460 |
|  | Cell viability [%] | 96.753 | 1.306 | 96.760 | 1.024 | 96.802 | 1.187 | 97.048 | 0.520 | 0.7438 | 0.6624 | 0.7578 |
|  | Mean diameter [µm] | 7.623 | 0.083 | 10.746 | 0.305 | 7.574 | 0.115 | 10.621 | 0.161 | 0.2160 | **< 0.0001** | 0.5938 |
|  | Cell survival [%] | 98.187 | 0.819 | 98.484 | 0.432 | 98.204 | 1.018 | 98.449 | 0.528 | 0.3279 | 0.9725 | 0.9243 |

**Supplementary Table 2.** Overview on clinical data at two time points from convalescent and non-infected, Covid-19-vaccinated athletes (Con N=7, Vac N=6). Data labelled pre was sampled at pre-season screening and data labelled post was sampled at post-quarantine from convalescent athletes and at next pre-season screening from vaccinated athletes. Data was analyzed using a 2x2 ANOVA with the factor infection status and time. Significant results (p<0.5) are highlighted in bold letters.

|  |  | **Con** | | | | **Vac** | | | |  |  |  |
| --- | --- | --- | --- | --- | --- | --- | --- | --- | --- | --- | --- | --- |
|  |  | *pre* | | *post* | | *pre* | | *post* | | p | | |
|  |  | Mean | SD | Mean | SD | Mean | SD | Mean | SD | *condition* | *Time* | *C:T* |
| **Cardiovascular** | |  |  |  |  |  |  |  |  |  |  |  |
|  | Systolic aortic pressure [mmHg] | 97.74 | 8.50 | 108.71 | 7.52 | 98.32 | 6.19 | 102.75 | 5.82 | 0.305 | **0.007** | 0.213 |
|  | Diastolic aortic pressure [mmHg] | 62.26 | 13.60 | 74.43 | 8.89 | 59.76 | 10.20 | 64.63 | 6.65 | 0.106 | **0.033** | 0.396 |
|  | HR brachial [Bpm] | 54.32 | 10.15 | 62.86 | 8.55 | 59.78 | 8.98 | 65.88 | 13.94 | 0.289 | 0.076 | 0.758 |
|  | HR radial [Bpm] | 54.71 | 8.86 | 62.14 | 9.23 | 59.63 | 8.82 | 65.63 | 13.77 | 0.284 | 0.093 | 0.854 |
|  | Systolic brachial pressure [mmHg] | 123.79 | 7.30 | 129.71 | 12.09 | 123.71 | 4.40 | 127.75 | 6.67 | 0.729 | 0.102 | 0.748 |
|  | Diastolic brachial pressure [mmHg] | 64.29 | 13.97 | 72.86 | 9.25 | 60.00 | 8.93 | 64.13 | 5.84 | 0.080 | 0.094 | 0.539 |
|  | Map Brachial [mmHg] | 79.05 | 13.94 | 91.81 | 7.92 | 76.27 | 7.68 | 85.33 | 5.78 | 0.180 | **0.003** | 0.587 |
| **Clinical chemistry** | |  |  |  |  |  |  |  |  |  |  |  |
|  | Leukocytes [cells/µl] | 6585.71 | 1463.36 | 5800.00 | 1226.10 | 5500.00 | 902.38 | 5812.50 | 1000.62 | 0.215 | 0.636 | 0.205 |
|  | Erytrocytes [cells/µl] | 4.81 | 0.34 | 4.89 | 0.34 | 5.00 | 0.31 | 4.94 | 0.17 | 0.279 | 1.000 | 0.538 |
|  | Hemoglobin [g/dl] | 14.73 | 0.70 | 14.69 | 1.03 | 14.70 | 0.91 | 14.63 | 0.80 | 0.889 | 0.851 | 0.960 |
|  | Haematocrit [%] | 42.29 | 2.14 | 42.43 | 2.76 | 42.50 | 2.83 | 42.75 | 1.49 | 0.759 | 0.818 | 0.951 |
|  | Thrombocytes [cells/l] | 210.71 | 47.84 | 235.57 | 54.49 | 205.25 | 37.50 | 212.50 | 50.20 | 0.421 | 0.382 | 0.618 |
|  | Retikolocytes [g/l] | 38.94 | 14.91 | 41.89 | 13.65 | 48.00 | 13.31 | 50.66 | 15.11 | 0.100 | 0.596 | 0.979 |
|  | HbA1c [%] | 5.36 | 0.27 | 5.43 | 0.27 | 5.21 | 0.28 | 5.13 | 0.35 | **0.049** | 0.903 | 0.470 |
|  | Na^+^ [mmol/l] | 140.00 | 1.15 | 139.71 | 1.25 | 140.00 | 1.69 | 140.00 | 1.51 | 0.788 | 0.801 | 0.788 |
|  | K^+^ [mmol/l] | 3.83 | 0.26 | 4.16 | 0.26 | 4.00 | 0.19 | 3.85 | 0.33 | 0.493 | 0.458 | **0.021** |
|  | Mg^-^ [mmol/l] | 0.83 | 0.04 | 0.82 | 0.03 | 0.77 | 0.04 | 0.82 | 0.04 | 0.093 | 0.210 | **0.043** |
|  | Cl^-^ [mmol/l] | 103.43 | 1.27 | 104.71 | 0.95 | 103.75 | 1.39 | 104.38 | 1.69 | 0.986 | 0.073 | 0.515 |
|  | Iron [µg/dl] | 120.00 | 32.32 | 140.29 | 24.64 | 125.00 | 43.29 | 132.38 | 52.77 | 0.922 | 0.373 | 0.666 |
|  | Ca^2+^ [mmol/l] | 2.37 | 0.10 | 2.33 | 0.05 | 2.33 | 0.09 | 2.35 | 0.11 | 0.703 | 0.838 | 0.304 |
|  | Phosphate [mg/dl] | 1.16 | 0.19 | 1.06 | 0.19 | 1.10 | 0.19 | 1.05 | 0.12 | 0.620 | 0.261 | 0.699 |
|  | Protein [g/l] | 72.43 | 6.53 | 70.14 | 4.34 | 68.38 | 2.50 | 71.63 | 3.07 | 0.420 | 0.674 | 0.090 |
|  | Urea [mg/dl] | 34.57 | 8.06 | 36.14 | 7.78 | 31.00 | 7.05 | 33.13 | 3.87 | 0.198 | 0.460 | 0.912 |
|  | Creatinine [mg/dl] | 1.07 | 0.21 | 0.90 | 0.08 | 0.98 | 0.05 | 1.03 | 0.07 | 0.737 | 0.216 | **0.014** |
|  | Cystatin C [mg/l] | 0.80 | 0.05 | 0.78 | 0.10 | 0.75 | 0.06 | 0.79 | 0.09 | 0.517 | 0.607 | 0.272 |
|  | GFR  [ml/min/1,73m^2^] | 91.91 | 23.66 | 105.81 | 9.24 | 100.41 | 7.44 | 94.49 | 8.64 | 0.778 | 0.508 | 0.056 |
|  | LDH [pg/µl] | 232.00 | 48.70 | 182.29 | 30.42 | 192.50 | 32.50 | 181.13 | 28.62 | 0.228 | **0.045** | 0.159 |
|  | GGT[U/l] | 22.00 | 4.82 | 21.43 | 9.73 | 19.13 | 7.32 | 17.50 | 5.53 | 0.216 | 0.667 | 0.845 |
|  | GOT [U/l] | 27.67 | 8.09 | 19.00 | 5.00 | 33.00 | 20.48 | 28.13 | 12.24 | 0.142 | 0.198 | 0.708 |
|  | GPT [U/l] | 29.33 | 6.95 | 33.14 | 16.97 | 37.25 | 37.45 | 28.25 | 9.19 | 0.871 | 0.695 | 0.449 |
|  | Bilirubin [µmol/l] | 1.17 | 1.16 | 0.87 | 0.64 | 1.25 | 0.71 | 1.21 | 0.52 | 0.441 | 0.596 | 0.656 |
|  | Cholesterol [mg/dl] | 153.86 | 29.41 | 173.29 | 20.85 | 165.38 | 30.91 | 168.50 | 34.90 | 0.760 | 0.332 | 0.461 |
|  | HDL [mg/dl] | 63.43 | 43.76 | 41.71 | 8.08 | 45.13 | 5.91 | 42.50 | 6.97 | 0.284 | 0.161 | 0.244 |
|  | LDL [mg/dl] | 87.57 | 29.04 | 124.86 | 20.53 | 105.88 | 28.88 | 112.00 | 31.42 | 0.792 | 0.053 | 0.140 |
|  | Triglycerides [mg/dl] | 135.71 | 74.49 | 110.57 | 34.39 | 103.25 | 46.20 | 136.25 | 49.11 | 0.862 | 0.763 | 0.144 |
|  | Ferritin [ng/dl] | 213.57 | 279.85 | 206.14 | 52.27 | 120.38 | 91.62 | 126.25 | 92.03 | 0.133 | 0.995 | 0.906 |
|  | Transferrin saturation [%] | 36.65 | 8.57 | 44.13 | 6.93 | 40.36 | 13.30 | 43.99 | 19.16 | 0.715 | 0.271 | 0.694 |
|  | Transferrin [g/dl] | 2.32 | 0.32 | 2.26 | 0.17 | 2.20 | 0.23 | 2.18 | 0.27 | 0.298 | 0.680 | 0.798 |
|  | CRP [mg/l] | 1.65 | 4.37 | 0.26 | 0.45 | 0.11 | 0.30 | 0.45 | 1.28 | 0.411 | 0.571 | 0.296 |
|  | CK [U/l] | 366.00 | 195.15 | 117.29 | 41.05 | 414.25 | 380.49 | 371.13 | 305.08 | 0.139 | 0.171 | 0.309 |
|  | Myoglobine [pg/ml] | 64.14 | 33.42 | 65.29 | 92.10 | 59.63 | 31.78 | 50.00 | 27.09 | 0.606 | 0.810 | 0.779 |
| **Hormones** | |  |  |  |  |  |  |  |  |  |  |  |
|  | TSH [mU/l] | 2.11 | 0.50 | 1.67 | 0.70 | 2.27 | 1.38 | 1.84 | 0.75 | 0.621 | 0.203 | 0.993 |
|  | fT4 [pmol/l] | 1.16 | 0.10 | 1.16 | 0.15 | 1.30 | 0.21 | 1.23 | 0.10 | 0.063 | 0.466 | 0.495 |
|  | fT3 [pmol/l] | 3.41 | 0.28 | 3.53 | 0.20 | 3.25 | 0.29 | 3.21 | 0.31 | **0.025** | 0.743 | 0.458 |
|  | 25 OH Vitamin D [ng/ml] | 34.24 | 6.46 | 27.57 | 15.50 | 30.04 | 12.49 | 30.61 | 8.39 | 0.888 | 0.500 | 0.386 |
|  | Parathyroid hormone [ng/l] | 35.83 | 19.62 | 26.10 | 10.68 | 25.50 | 5.64 | 22.54 | 4.69 | 0.108 | 0.153 | 0.424 |
|  | BNP [ng/l] | 25.86 | 20.46 | 17.29 | 27.23 | 12.38 | 9.29 | 9.00 | 6.97 | 0.100 | 0.371 | 0.687 |
|  | Testosterone [µg/l] | 569.65 | 113.34 | 551.33 | 123.46 | 570.19 | 116.53 | 494.48 | 109.44 | 0.531 | 0.255 | 0.513 |
